# Biodiesel and flavor compound production using a novel promiscuous cold-adapted SGNH-type lipase (*Ha*SGNH1) from the psychrophilic bacterium *Halocynthiibacter arcticus*

**DOI:** 10.1186/s13068-020-01696-x

**Published:** 2020-03-16

**Authors:** Ly Thi Huong Luu Le, Wanki Yoo, Sangeun Jeon, Changwoo Lee, Kyeong Kyu Kim, Jun Hyuck Lee, T. Doohun Kim

**Affiliations:** 1 Department of Chemistry, College of Natural Science, Sookmyung Women’s University, Seoul, 04310 South Korea; 2 Department of Molecular Cell Biology, Samsung Biomedical Research Institute, Sungkyunkwan University School of Medicine, Suwon, 440-746 South Korea; 3 Department of Polar Sciences, University of Science and Technology (UST), Incheon, 21990 South Korea; 4 Unit of Polar Genomics, Korea Polar Research Institute (KOPRI), Incheon, 21990 South Korea

**Keywords:** *Ha*SGNH1, *Halocynthiibacter arcticus*, Immobilization, SGNH-type lipase, Substrate specificity, Biodiesel

## Abstract

**Background:**

Biodiesel and flavor compound production using enzymatic transesterification by microbial lipases provides mild reaction conditions and low energy cost compared to the chemical process. SGNH-type lipases are very effective catalysts for enzymatic transesterification due to their high reaction rate, great stability, relatively small size for convenient genetic manipulations, and ease of immobilization. Hence, it is highly important to identify novel SGNH-type lipases with high catalytic efficiencies and good stabilities.

**Results:**

A promiscuous cold-adapted SGNH-type lipase (*Ha*SGNH1) from *Halocynthiibacter arcticus* was catalytically characterized and functionally explored. *Ha*SGNH1 displayed broad substrate specificity that included *tert*-butyl acetate, glucose pentaacetate, and *p*-nitrophenyl esters with excellent stability and high efficiency. Important amino acids (N83, M86, R87, F131, and I173F) around the substrate-binding pocket were shown to be responsible for catalytic activity, substrate specificity, and reaction kinetics. Moreover, immobilized *Ha*SGNH1 was used to produce high yields of butyl and oleic esters.

**Conclusions:**

This work provides a molecular understanding of substrate specificities, catalytic regulation, immobilization, and industrial applications of a promiscuous cold-adapted SGNH-type lipase (*Ha*SGNH1) from *H. arcticus*. This is the first analysis on biodiesel and flavor synthesis using a cold-adapted halophilic SGNH-type lipase from a *Halocynthiibacter* species.

## Background

**Fig. 1:**
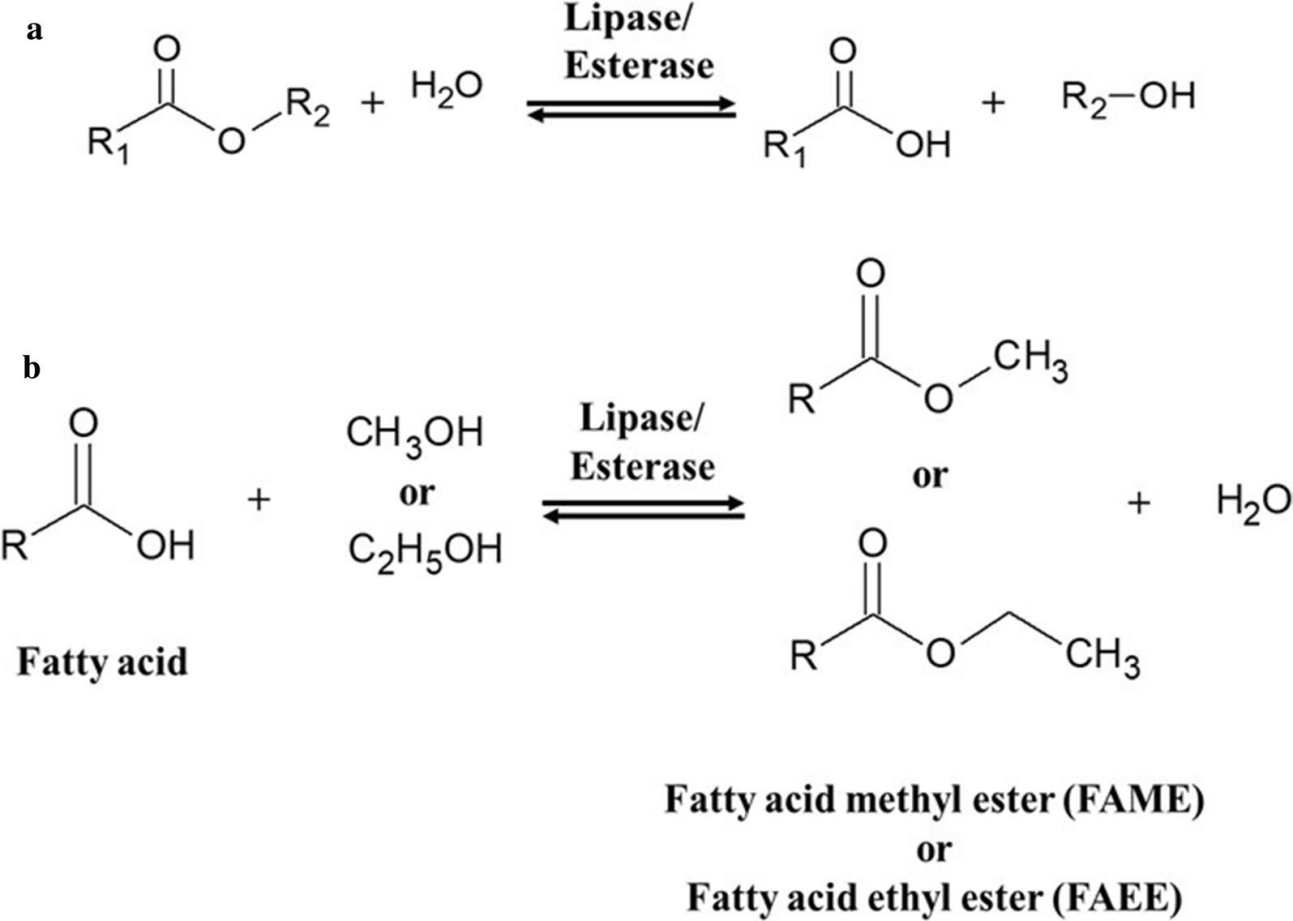
Schematic reaction diagram of lipase/esterase. **a** In a general reaction, lipase/esterase are involved in the hydrolysis and formation of esterase. **b** Biofuels such as fatty acid methyl ester (FAME) or fatty acid ethyl ester (FAEE) can be prepared using lipase/esterase with fatty acid and methanol or ethanol

Lipolytic enzymes such as lipases, esterases, and phospholipases catalyze the hydrolysis and transesterification of ester-containing compounds. Although these enzymes are widely distributed across the three domains of the tree of life, microbial enzymes have been extensively exploited in the fine chemical, pharmaceutical, bioenergy, pulp and paper, and food industries [ [1–3], see Fig. 1]. In addition, these enzymes have been identified as potential biocatalysts for biodiesel synthesis due to their excellent stability and high efficiency [4, 5]. They share similar structural and catalytic features including a consensus sequence around the catalytic center, a conserved catalytic triad (Ser-Asp/Glu-His), broad substrate specificity, and a lack of essential cofactors [6]. Lipase (EC 3.1.1.3, triacylglycerol hydrolase) is mainly active against water-insoluble substrates such as long-chain triglycerides while esterase (EC 3.1.1.1, carboxyl ester hydrolase), hydrolyzes short chain triglycerides and simple water-soluble esters. In addition to their different substrate specificity, lipases can be distinguished from esterases by their specific phenomenon of interfacial activation [3, 6].

Bacterial lipases/esterases are classified into eight families (I to VIII) [7]. Among them, SGNH-type lipases (family II) are characterized by the presence of four conserved blocks of I–III and V [8]. In these enzymes, the catalytic serine is located in the highly conserved motif Gly-Asp-Ser-Leu (GDSL) in the N-terminal region. In addition, Gly and Asn in motif II and III are involved in the formation of an oxyanion hole, and His in motif V is the general acid–base in the catalysis. Cold-adapted lipolytic enzymes of psychrophilic microorganisms have attracted much interest [9, 10]. These enzymes display high activity at low temperatures compared to other lipases from different environments. To date, a number of cold-adapted lipases have been identified and characterized from microorganisms [10], but there are very few reports of SGNH-type lipases from psychrophilic bacteria [11–13]. In addition, there is very little information on biodiesel and flavor production using these cold-adapted SGNH-type lipases.

Here, a novel promiscuous cold-adapted SGNH-type lipase (*Ha*SGNH1) was cloned from the psychrophilic bacterium *Halocynthiibacter arcticus*, which was isolated from Arctic marine sediment [14]. The genome of *H. arcticus* NCBI Reference Sequence: NZ_CP007142) was fully sequenced, providing a rich resource of new enzymes that potentially function at low temperatures and high salt concentrations [15]. To our knowledge, no SGNH-type lipase has previously been identified from *H. arcticus*. The recombinant enzyme was purified, biochemically characterized, mutated using site-directed mutagenesis, and immobilized for the synthesis of butyl and oleic esters. This study is the first example of biodiesel and flavor compound production using a cold-adapted SGNH-type lipase.

## Results and discussion

### *Bioinformatic analysis of Ha*SGNH1

**Fig. 2:**
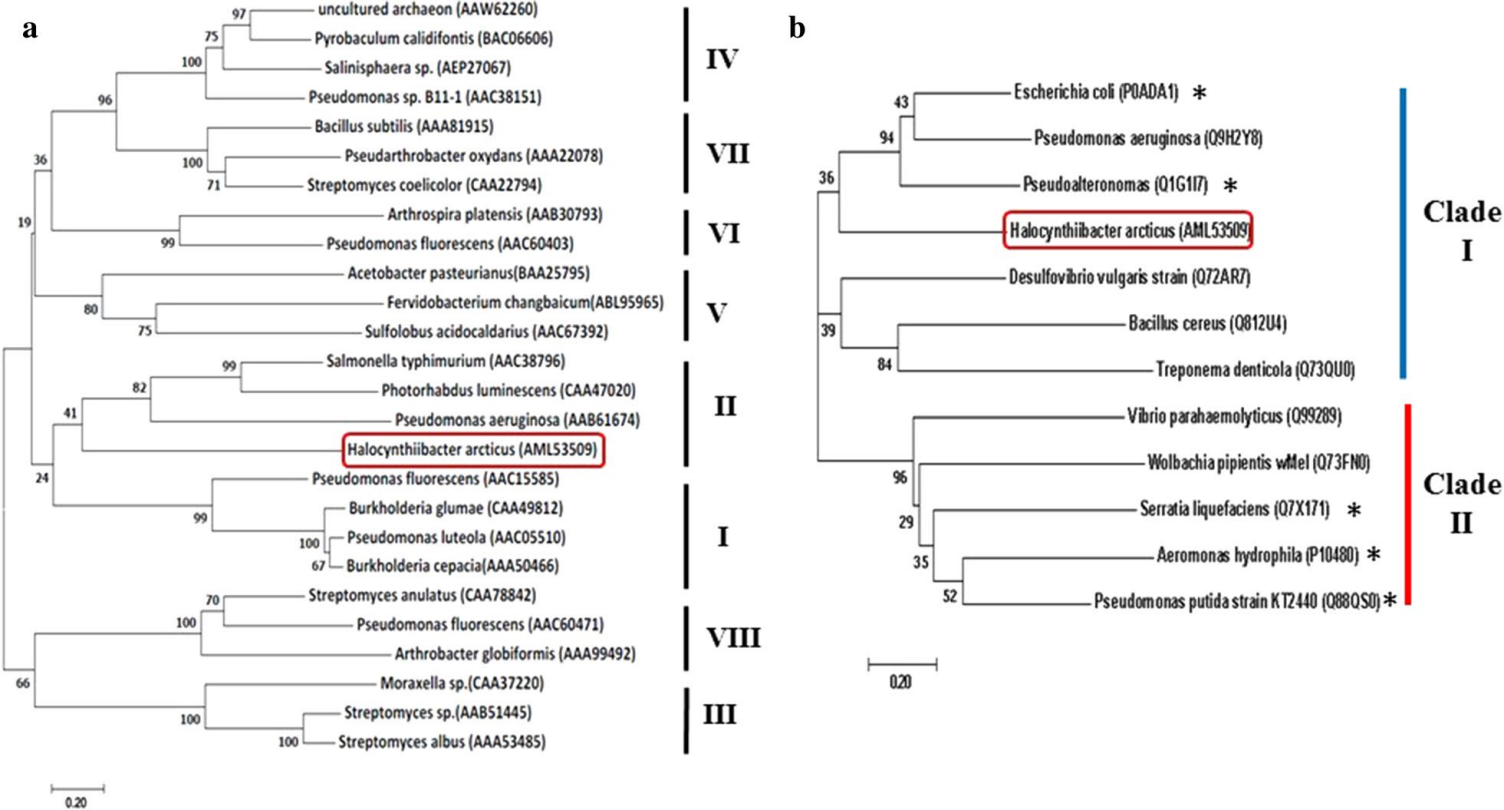
Phylogenetic tree analysis of *Ha*SGNH1. Phylogenetic analysis of *Ha*SGNH1 with bacterial lipases/esterases family I–VIII (**a**), and clade I and II of GDSL family (**b**). The location of *Ha*SGNH1 in the phylogenetic tree is indicated by a red box. The phylogenetic trees were constructed with MEGA v7.0 using the neighbor-joining method and all sequences were retrieved from the NCBI database. Biochemically characterized proteins were shown with *

A gene encoding a novel SGNH-type lipase (*Ha*SGNH1, locus tag: WP_082802169) was identified on the *H. arcticus* chromosome using in silico bioinformatic analysis. Sequence analysis revealed that *Ha*SGNH1 has a molecular mass of ~ 25.3 kDa and consists of a single 232 amino acid polypeptide chain with a pI of 4.31. No secretory signaling peptides were detected in the sequence. *Ha*SGNH1 shared the highest sequence identity with an arylesterase from *Oceanicola litoreus* (53% identity, WP_074257955), followed by esterase TesA from *Confluentimicrobium lipolyticum* (51% identity, SMX44997), an acyl-CoA thioesterase-1 from *O. litoreus (*50% identity, SIO30670), and an arylesterase from *Pacificibacter maritimus* (50% identity, SIO30670). However, there have been no studies of these proteins, and their relevant properties are largely unknown. For phylogenetic tree analysis, 26 lipases from eight (I–VIII) bacterial families were investigated using the neighbor-joining method. The results indicated that *Ha*SGNH1 might belong to family II lipases/esterases (Fig. 2a), which is grouped into two subfamilies of clade I and clade II [8, 16]. As shown in Fig. 2b, *Ha*SGNH1 was clustered in the clade I subfamily with a lysophospholipase A (TesA) from *Pseudomonas aeruginosa* (Q9HZY8).

**Fig. 3:**
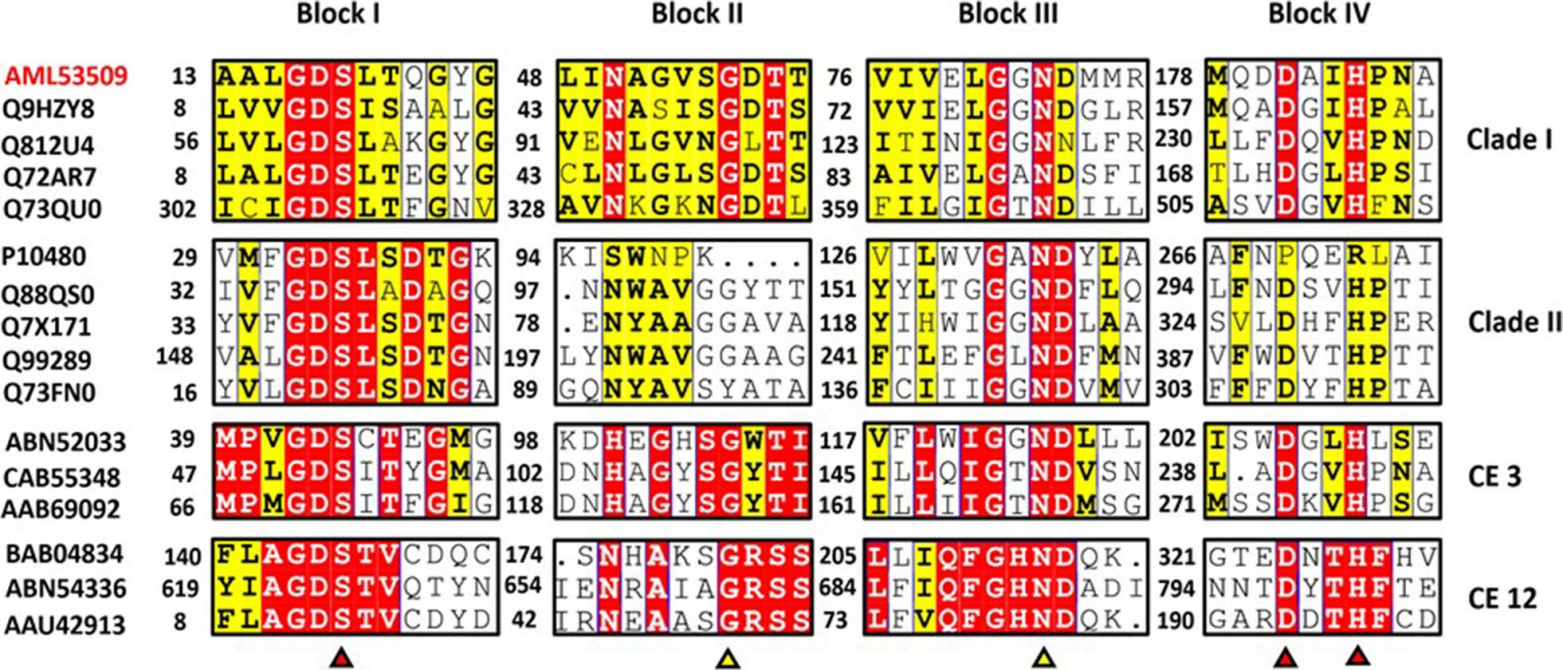
Multiple sequence alignments of *Ha*SGNH1. Sequence alignment of *Ha*SGNH1 (AML53509) with related proteins from SGNH clade I, II, and carbohydrate esterase families 3 (CE3) and 12 (CE12). Highly conserved sequences including the catalytic triad, glycine, and asparagine are shown as red and yellow triangles

As shown in Fig. 3, four blocks (I, II, III, and V) of *Ha*SGNH1 are highly conserved based on multiple sequence alignments among the clade I and clade II subfamilies. Ser^138^ in block I and Asp^216^ and His^244^ in block V form the catalytic triad, with Ser^18^ located in a highly conserved GDSL tetrapeptide motif. Among currently known 16 carbohydrate esterase (CE) families [17], *Ha*SGNH1 is highly homologous to CE family members of III (CE3) and XII (CE12). Interestingly, significant degrees of conservation in protein length and catalytic amino acid residues suggest that there may a common ancestral enzyme shared by all of these enzymes. *Ha*SGNH1 has a high proportion of small amino acids like Gly (7.8%) and Ala (8.6%) based on sequence analysis. In addition, low levels of proline and Arg/ (Arg + Lys) are also found in *Ha*SGNH1. Moreover, *Ha*SGNH1 contains a higher percentage of acidic (Asp and Glu, 13.4%) than basic (Lys and Arg, 6.5%) amino acids, which is considered to be an important property observed in psychrophilic enzymes [18, 19].

### Characterizations of *Ha*SGNH1

**Fig. 4:**
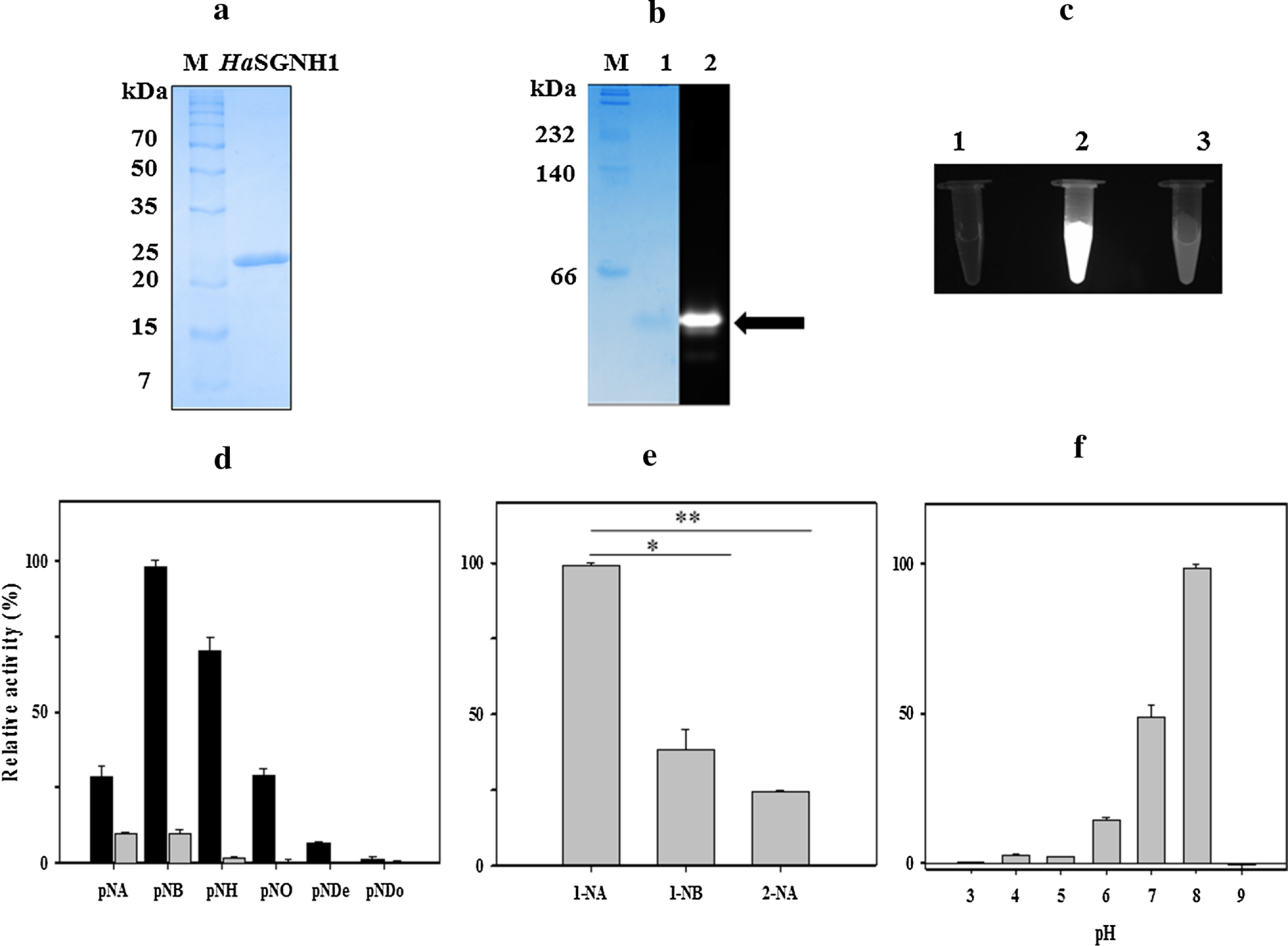
Characterization of *Ha*SGNH1. **a** SDS-PAGE analysis of *Ha*SGNH1. **b** Native-PAGE analysis and zymographic analysis of *Ha*SGNH1. After running native-PAGE, the resulting gel was stained with Coomassie Brilliant Blue R-250 (*lane 1*) or and 4-methylumbelliferone (4-MU) acetate (*lane 2*). **c** Hydrolysis of 4-methylumbelliferyl (4-MU) acetate and phosphate. *Ha*SGNH1 only (*lane 1*), 4-MU acetate with *Ha*SGNH1 (*lane 2*), and 4-MU phosphate with *Ha*SGNH1 (*lane 3*). Hydrolysis of 4-MU acetate in an Eppendorf tube containing *Ha*SGNH1 was observed as strong fluorescence after UV illumination at 254 nm. **d** The hydrolysis of *p*-nitrophenyl ester derivatives by wild-type *Ha*SGNH1 (black) and its S18A variant (gray). *p*NA: *p*-nitrophenyl acetate; *p*NB: *p*-nitrophenyl butyrate; *p*NH: *p*-nitrophenyl hexanoate; *p*NO: *p*-nitrophenyl octanoate; *p*NDe: *p*-nitrophenyl decanoate; *p*NDo: *p*-nitrophenyl dodecanoate. **e** The hydrolysis of naphthyl ester derivatives by *Ha*SGNH1. The symbols * and ** represent significant differences (*p*-value < 0.001 (*) or *p*-value < 0.0001 (**), respectively), determined by Student’s *t* tests. (F) pH stability of *Ha*SGNH1. *Ha*SGNH1 was incubated at different pH between 3 and 10. Hydrolase activities were determined by measuring the amount of *p*-nitrophenol released during hydrolysis at 405 nm using a VersaMax 680 microplate reader (Bio-Rad Laboratories, CA, USA). In these experiments, the enzyme activity of *Ha*SGNH1 under standard assay condition was defined as 100%

Recombinant *Ha*SGNH1 was purified to near homogeneity using an immobilized Ni^2+^-affinity column. The molecular mass of *Ha*SGNH1 was estimated to be ~ 24 kDa using SDS-PAGE (Fig. 4a), which is similar to the mass of estSL from *Alkalibacterium* sp. SL3 [11] and EstA from *Pseudoalteromonas* sp. 643A [12]. However, it is smaller than the mass of a cold-adapted 36 kDa GDSL family esterase from *Photobacterium* sp. J15 [13]. *Ha*SGNH1 behaves as a monomeric conformant in the gel filtration columns (Additional file 1: Fig. S1A), which is similar to *Nm*SGNH1 from *Neisseria meningitides* [20]. However, several other SGNH family esterases reportedly exist in an oligomeric conformation [21, 22]. To identify in gel esterase activity of the enzyme, 4-methylumbelliferone (4-MU) acetate was added to an PAGE gel, an artificial substrate which is known to be cleaved by esterases to acetate and to the fluorescent compound 4-MU. As shown in Fig. 4b, strong fluorescence was detected, by activity staining using 4-MU acetate, at the position where purified *Ha*SGNH1 was located. Furthermore, strong fluorescence was observed for 4-MU acetate and *Ha*SGNH1 but not for 4-MU phosphate and *Ha*SGNH1 (Fig. 4c). The far-ultraviolet (UV) circular dichroism (CD) spectrum of *Ha*SGNH1 showed a characteristic shape of negative ellipticity at approximately 210 - 220 nm (Additional file 1: Fig. S1B), which is usually observed in SGNH-type lipases [23]. Thermal denaturation of *Ha*SGNH1 was examined by monitoring the CD signal at 222 nm from 15 °C to 80 °C. *Ha*SGNH1 showed very few changes up to 25 °C, and the melting temperature was determined to be 42 °C (Additional file 1: Fig. S1C).

The hydrolytic activity of *Ha*SGNH1 was analyzed using *p*-NP ester substrates of different chain lengths. *p*-NP esters substrates were hydrolyzed by lipases/esterases and the resulting product of *p*-nitrophenol was measured at 405 nm. As shown in Fig. 4d, *Ha*SGNH1 had a strong substrate preference for *p*-nitrophenyl acetate (*p*-NA, C_2_) or *p*-nitrophenyl hexanoate (*p*-NH, C_6_), and the highest activity with *p*-nitrophenyl butyrate (*p*-NB, C_4_). Additionally, very little activity was observed for the long-chain substrates *p*-NDe (C_10_), *p*-NDo (C_12_), or *p*-nitrophenyl phosphate (p-NPP). This preference for short-chain *p*-NP esters was also observed for other SGNH-type family members like Cbes-AcXE2 [24] or EstL5 [25]. However, Ser^29^ mutation abolished most of the hydrolytic activity for *p*-NP esters. In addition, naphthyl esters could be hydrolyzed by lipases/esterases and the resulting product of naphthol could be measured at 310 nm. When naphthyl esters were used as substrates, the highest *Ha*SGNH1 activity was observed with 1-naphthyl acetate (1-NA), followed by 2-naphthyl acetate (2-NA) (Fig. 4e). *Ha*SGNH1 showed regioselectivity, exhibiting only 25% activity toward 2-NA compared to 1-NA.

*Ha*SGNH1 displayed its optimal activity at a weakly basic pH of 8.0, whereas 65% of its optimal activity was observed at pH 7.0 (Fig. 4f). This is similar to most other SGNH-type lipases like *Nm*SGNH1 [20], Sm23 [21], and LI22 [26]. Furthermore, *Ha*SGNH1 retained about 65% of its initial activity in the presence of 10% ethanol and about 30% of its activity in the presence of 0. 1% Triton X-100 as shown in Additional file 1: Fig. S1D. In contrast, the addition of 0.1% SDS resulted in complete loss of *Ha*SGNH1 activity, which is often observed in cold-active lipases like Lip2Pc [27] or EstPc [28]. *Ha*SGNH1 was also stable in high NaCl and glycerol concentrations. This is reminiscent of the halophilic property of *H.* *arcticus*, which showed optimal growth at 2.0 % NaCl [14]. Interestingly, *Ha*SGNH1 was highly active in the absence of NaCl, unlike most halophilic enzymes (Additional file 1: Fig. S1E). Specifically, *Ha*SGNH1 retained ~ 115% of its activity in the presence of 0.5 to 1.0 M NaCl. Moreover, *Ha*SGNH1 retained about 50% of its original activity in 2.0 M NaCl and it exhibited maximum activity in 20% glycerol. However, very little activity was detected in 0.5 M urea concentrations (Additional file 1: Fig. S1F).

**Fig. 5:**
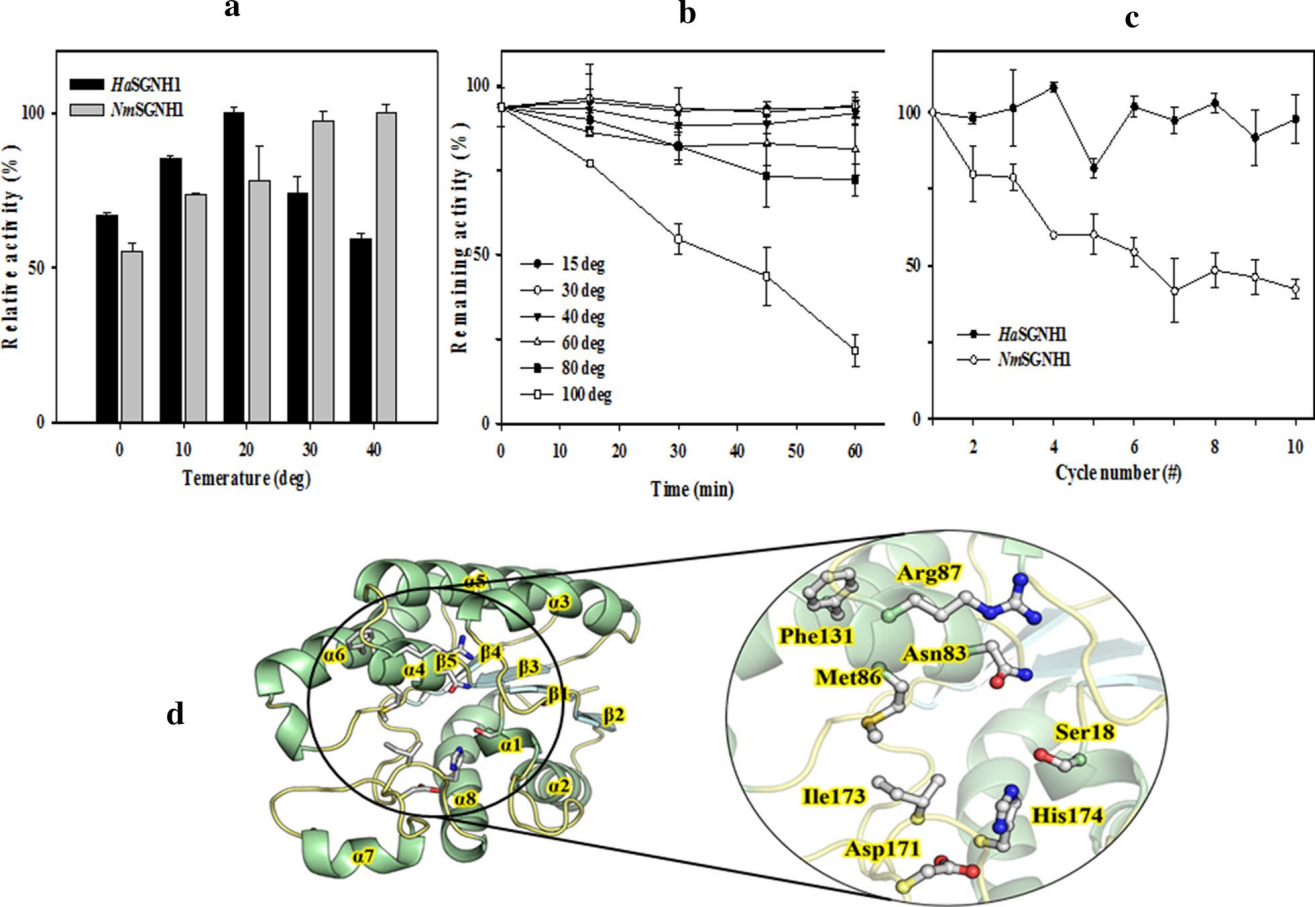
Cold-adapted properties and structural model of *Ha*SGNH1. **a** Optimum temperature of *Ha*SGNH1 and *Nm*SGNH1. **b** Freeze-and-thaw experiment with *Ha*SGNH1 and *Nm*SGNH1. **c** Thermal stability of *Ha*SGNH1 from 15 to 100 °C. In these experiments, the enzyme activity of *Ha*SGNH1 under standard assay condition was defined as 100%. **d** A molecular model of *Ha*SGNH1 was shown in ribbon diagram. Catalytic triad and the residues critical for the hydrolysis are shown as a ball-and-stick model in a large circle

As shown in Fig. 5a, the optimal temperature of *Ha*SGNH1 was about 20 °C, which is comparable to estSL3 [11], slightly lower than estS9 [29] and EstA [30], and higher than a GDSL family esterase from *Photobacterium sp.* J15 [13]. In addition, *Ha*SGNH1 exhibited high relative activities at low temperatures, retaining ~ 70% of its maximum activity even at 0 °C. The value is higher than other cold-adapted SGNH-type lipases like estSL3 [11], estS9 [29], or EstA [30]. *Ha*SGNH1 thermostability was investigated over a temperature range from 15 to 100 °C (Fig. 5b). *Ha*SGNH1 enzyme activity did not change notably after a 1 h incubation at 60 °C. *Ha*SGNH1 activity gradually decreased at 80 °C; about 70% of the initial activity was retained after 1 h. Even at 100 °C, less than 50% enzyme activity was lost after 30 min, which is higher than most other cold-adapted lipases [9]. *Ha*SGNH1 stability at cold temperatures was analyzed using freeze–thaw cycles. As shown in Fig. 5c, most of its initial activity was maintained even after 9 cycles, suggesting that *Ha*SGNH1 was highly stable at cold temperatures. High activity and excellent thermostability could make *Ha*SGNH1 a great candidate for industrial applications, such as heat-sensitive chemical synthesis.

The hydrolytic properties of *Ha*SGNH1 for tertiary alcohol esters (TAEs) and lipids were also studied using a colorimetric assay with phenol red [31]. TAEs included *tert*-butyl acetate, α-terpinyl acetate, and linalyl acetate. All these substrates were used for hydrolysis and the released acetic acid could lead to the color change of the solution. As shown in Additional file 1: Fig. S2A, *Ha*SGNH1 could effectively hydrolyze *tert*-butyl acetate, but not linalyl acetate or α-terpinyl acetate. Additionally, significant *Ha*SGNH1 hydrolytic activity was detected only for glyceryl tributyrate, indicated by the yellow color of the solution (Additional file 1: Fig. S2B).

*Ha*SGNH1 exhibited hyperbolic kinetic behavior with three different substrates (*p*-NA, *p*-NB, and *p*-NH) (Additional file 1: Fig. S3A-C). The maximum velocity (V_max_), turnover number (*k*_cat_), Michaelis–Menten constant (*K*_*M*_), and catalytic efficiency (*k*_cat_*/K*_*m*_) were determined using the Michaelis–Menten model. The *V*_max_ and *K*_*M*_ of *Ha*SGNH1 with *p*-NB as a substrate are 1.00 s^−1^μM and 0.80 mM, respectively (Table 1). Catalytic efficiency shares the same trend towards different chain lengths of *p*-nitrophenyl esters (see also Fig. 4d). From these parameters, the catalytic efficiency for *p*-NB (21.3 s^−1^ mM^−1^) was about 2.5-fold higher than *p*-NA (8.9 s^−1^ mM^−1^), indicating that *Ha*SGNH1 works more efficiently on *p*-NB than *p*-NA. Although the substrate specificity of *Ha*SGNH1 toward *p*NP esters is similar to *Nm*SGNH1, the catalytic efficiency of *Ha*SGNH1 for *p*-NB is higher than *Nm*SGNH1 [20]. These characteristics may make *Ha*SGNH1 extremely useful as a biocatalyst for industrial applications (Table 2).

**Table 1:** Kinetic parameters of *Ha*SGNH1 towards *p*-NP esters

Substrate	Vmax (s^−1^ μM)	*K*_M (_mM)	*k*_cat_ (s^−1^)	*k*_cat_/*K*_M (_s^−1^ mM)
*p*-NA (C_2_)	0.49 (± 0.03)	0.93 (± 0.15)	8.38 (± 0.53)	8.9
*p*-NB (C_4_)	1.00 (± 0.11)	0.80 (± 0.23)	17.1 (± 1.83)	21.3
*p*-NH (C_6_)	0.96 (± 0.08)	0.99 (± 0.20)	16.5 (± 1.45)	16.7

Kinetic parameters of *Ha*SGNH1 were determined using Michaelis–Menten kinetics. Three substrates of *p*-NP esters *(p*-NA, *p*-NB, and *p*-NH) were used for comparison. Kinetic parameters (*V*_max_, *K*_*M*_, and *k*_cat_) were calculated by directly fitting to the Michaelis–Menten plot

**Table 2:** Molecular characteristics of *Ha*SGNH1 with other SGNH-type lipolytic enzymes

Microorganism	Protein	Mass (kDa)	Oligomeric state	Optimal temp/pH	Detergents stability	NaCl stability	Refs.
*Halocynthiibacter arcticus*	*Ha*SGNH1	24	Monomer	20°C/8.0	No	Yes	This study
*Alkalibacterium* sp.	estSL3	25	n.e.	30°C/8.0	Yes	Yes	[11]
*Pseudoalteromonas* sp.	EstA	23	Monomer	35°C/10.0	Yes	Yes	[12]
*Photobacterium* sp.	J15	36	n.e.	20°C/8.0	Yes	n.e.	[13]
*Neisseria meningitides*	NmSGNH1	21	Monomer	n.e/8.0	No	n.e.	[20]
*Sinorhizobium meliloti*	Sm23	23	Oligomer	n.e./8.0	No	n.e.	[23]

*n.e.* not explained

### Homology modeling and mutagenesis of HaSGNH1

A structural model of *Ha*SGNH1 was constructed based on the crystal structures of three homologous proteins with high sequence identity: a GDSL-esterase from *Pseudoalteromonas* sp. 643A (PDB code: 3HP4), TesA from *Pseudomonas aeruginosa (*PDB code: 4JGG), and thioesterase I (TAP) from *Escherichia coli* (1JRL). The structural model of *Ha*SGNH1 consists of five central parallel β-sheets enclosed by two layers of four α-helices per layer (Fig. 5d). The putative catalytic triad of Ser^18^, Asp^171^, and His^174^ are positioned close to the surface within a catalytic pocket (Fig. 5d, *circled region*). The promiscuous substrates of *Ha*SGNH1 could be explained by the presence of a widely solvent-exposed substrate-binding pocket [18, 23]. As shown in Additional file 1: Fig. S2C, the substrate binding pocket was mainly surrounded by the five amino acids Asn^83^, Met^86^, Arg^87^, Phe^131^, and Ile^173^, that may control the entrance of substrates via noncovalent interactions. In addition, similar amino acids were also observed in other SGNH-type lipases like EstA [12] and TesA [32], as shown in Additional file 1: Fig. S2D–E.

**Fig. 6:**
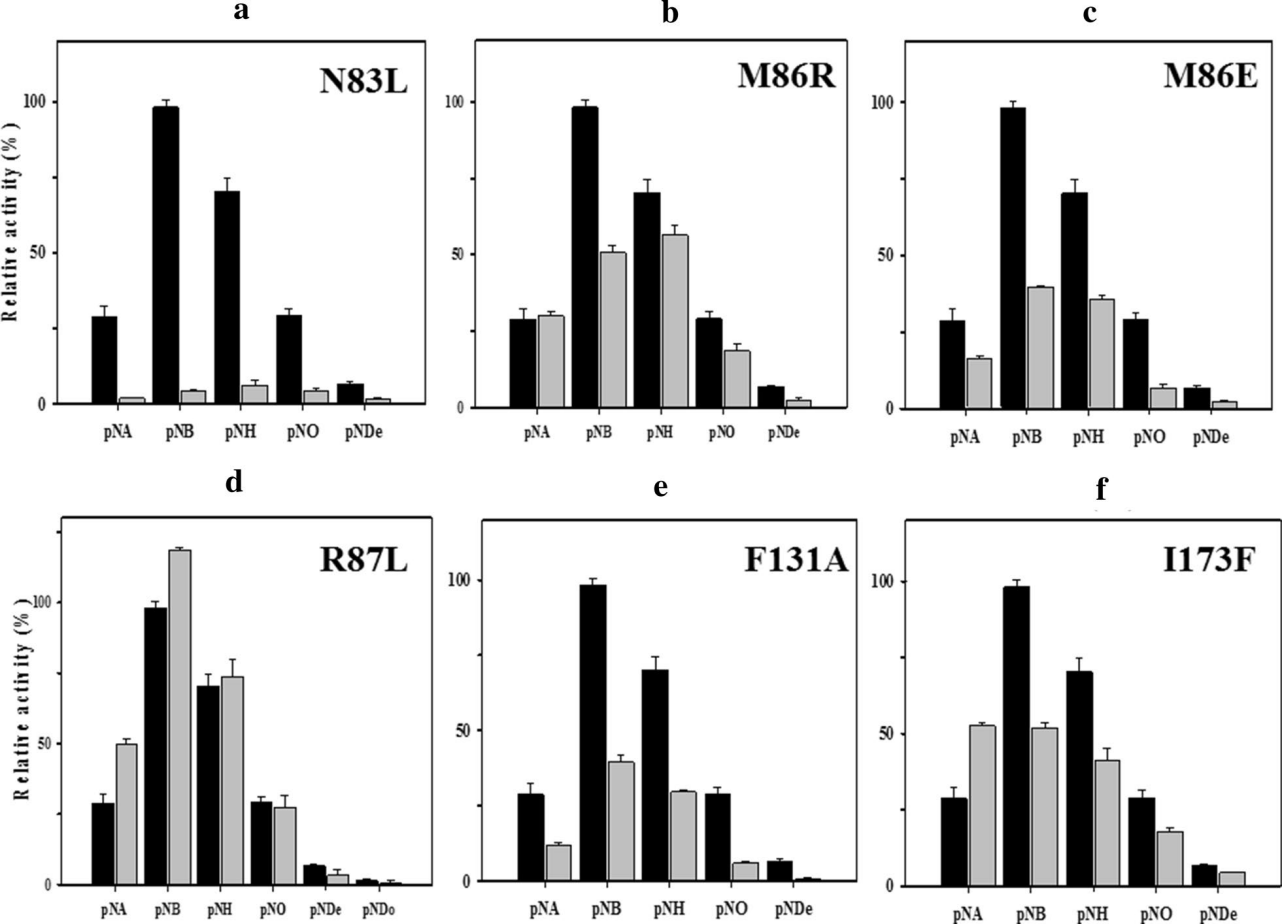
Substrate specificity of *Ha*SGNH1 variants. Substrate specificity of *Ha*SGNH1 variants toward *p*-nitrophenyl esters. Results of **a** N83L, **b** M86R, **c** M86E, **d** R87L, **e** F131A, and **f** I173F are shown. Results from wild-type *Ha*SGNH1 are shown for comparison in black. In these experiments, the enzyme activity of wild-type *Ha*SGNH1 under standard assay condition was defined as 100%. All experiments were performed at least in triplicate

**Fig. 7:**
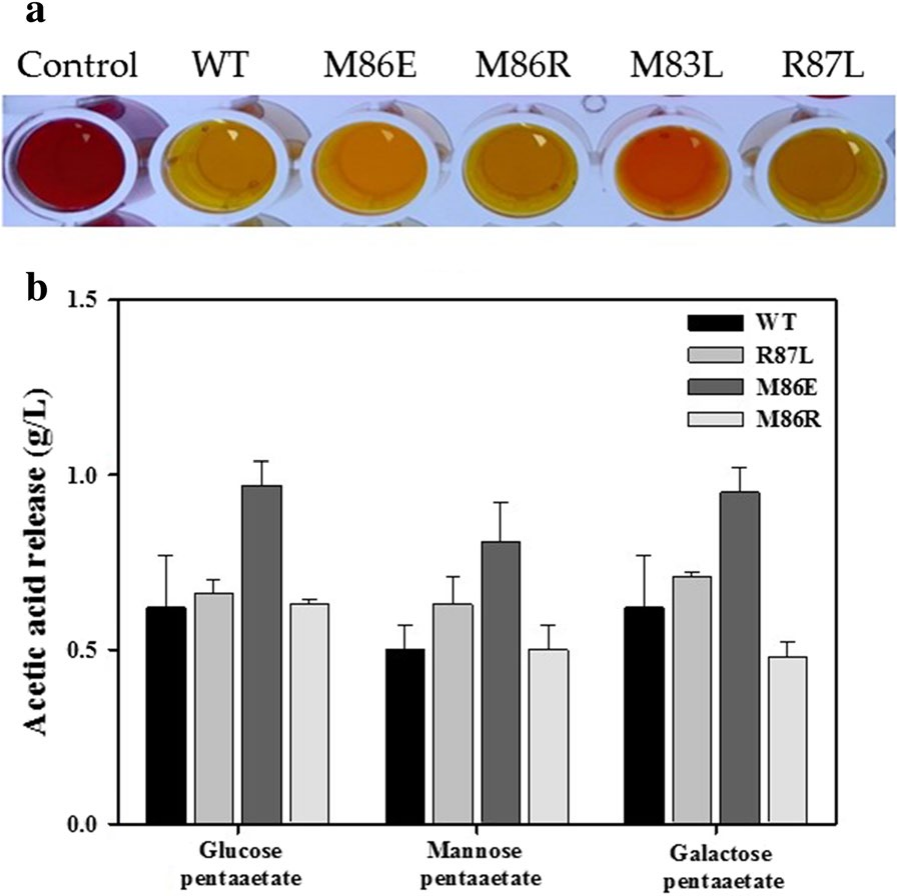
Colorimetric assay and released acid determination. **a** The pH indicator-based colorimetric assay was performed to verify the activity of *Ha*SGNH1 and its variants toward glyceryl tributyrate. Hydrolysis of glyceryl tributyrate was monitored by color changes from red to yellow due to a pH drop caused by released acid [31]. **b** The amount of acetic acid released from the hydrolysis of carbohydrate acetates by *Ha*SGNH1 and its variants. Quantifications of released acetic acid from acetylated substrates were determined using an acetic acid kit following the manufacturer’s instructions (K-ACET, Megazyme, USA)

To investigate the importance of these amino acid residues, six variants of enzymes (N83L, M86E, M86R, R87L, F131A, and I173F) were constructed using site-directed mutagenesis. After expression and purification, the catalytic activities and substrate specificities of these variants were studied and compared to wild-type *Ha*SGNH1. As expected, the activity of N83L, which is a part of block III, toward *p*-NP esters was almost lost (Fig. 6a, see also Fig. 3). The activity of M86E and M86R toward *p*-NP esters decreased compared to that of wild-type *Ha*SGNH1 (Fig. 6B, ANOVA, *p *< 0.05). However, R87L exhibited significantly enhanced activities, while F131A and I173F had a reduced level of hydrolytic activity towards *p*-NB compared to wild-type *Ha*SGNH1 (Fig. 6c–e, ANOVA, *p* < 0.05). Specifically, R87L exhibited 120% activity for *p*-NB, while M86R and M86E retained only about 50% and 40% of the enzyme activity of wild-type *Ha*SGNH1, respectively. In addition, F131A and I173F retained only about 45% and 50% of the enzyme activity of wild-type *Ha*SGNH1. Interestingly, M86R and I173F showed substantial changes in substrate specificity. M86R could more easily accept a larger substrate like *p*-NH, while I173F showed high activity for *p*-NA. In I173F, the bulky nature of Phe side chain may help facilitate enhanced binding of the shorter chain fatty acid substrate (Additional file 1: Fig. S2C). Similar behavior was also observed for naphthyl ester derivatives (data not shown). As shown in Fig. 7a, only N83L could not effectively hydrolyze glyceryl tributyrate (GTB). Furthermore, M86E showed enhanced activity for carbohydrate acetates like glucose pentaacetate (Fig. 7b, ANOVA, *p* < 0.05).

While the catalytic efficiency (*k*_cat_/*K*_*m*_) of R87L was higher than wild-type *Ha*SGNH1, M86R and M86E showed drastically reduced catalytic efficiency values. The *k*_cat_ values were very similar for these two variants, ranging from 4.0 to 5.1 s^−1^, compared to 17.1 s^−1^ for wild-type *Ha*SGNH1 (Table 3 and Additional file 1: Fig. S3D–F). With relatively small changes of *K*_*m*_, the reduction in catalytic efficiency resulted from small *k*_cat_ values. Collectively, these mutations in the substrate-binding region could control the catalytic activity, substrate specificity, and kinetic parameters of *Ha*SGNH1. Further structural studies are required to tailor *Ha*SGNH1 for biotechnological applications.

**Table 3:** Kinetic parameters of *Ha*SGNH1 and its variants toward *p*-NB

Substrate	Vmax (s^−1^ μM)	*K*_M_ (mM)	*k*_cat_ (s^−1^)	*k*_cat_/*K*_M (_s^−1^mM)
Wild type	1.00 (± 0.11)	0.80 (± 0.23)	17.1 (± 1.83)	21.3
R87L	1.15 (± 0.06)	0.68 (± 0.10)	15.0 (± 0.84)	22.2
M86R	0.78 (± 0.05)	1.26 (± 0.17)	5.1 (± 0.35)	4.2
M86E	0.61 (± 0.06)	1.06 (± 0.23)	4.0 (± 0.39)	3.8

Kinetic parameters of *Ha*SGNH1 and its four variants were determined using *p*-NB. Kinetic parameters (*V*_max_, *K*_*M*_, and *k*_cat_) were calculated by directly fitting to the Michaelis-Menten plot

### Immobilization of *Ha*SGNH1

Although multiple strategies have been explored to promote the use of enzymes as biocatalysts, enzyme immobilization is one of the most widely accepted methods due to low cost, fast recovery, and high product yields [33, 34]. In previous reports, immobilized SGNH-type lipases were reported to have improved thermal stability, better tolerance to organic solvents, and higher pH stability than free enzymes, which are associated with reduced conformational flexibility and thermal vibrations [20, 22, 26]. Here, we tried to enhance the potential industrial applicability of *Ha*SGNH1 using immobilization via covalent attachment and crosslinking approaches. First, *Ha*SGNH1-CLEAs were prepared by precipitating *Ha*SGNH1 with ammonium sulfate and glutaraldehyde [35]. As shown in Additional file 1: Fig. S4A, SEM images of *Ha*SGNH1-CLEAs were observed as globular-like structures with an average particle size of 0.1 μm. The operational stability of *Ha*SGNH1-CLEAs was studied for up to ten reuse cycles. Interestingly, more than 55% of the initial activity was still observed even after the tenth cycle (Additional file 1: Fig. S4A).

Recently, addition of arginine was shown to increase the stability of CLEA-immobilized enzymes [36]. In Additional file 1: Fig. S4B, SEM images of *Ha*SGNH1-Arg-CLEAs show bulbs, with an average size of 0.1 to 0.2 μm. The operational stability of *Ha*SGNH1-Arg-CLEAs was studied over 10 cycles. As shown in Additional file 1: Fig. S4B, *Ha*SGNH1-Arg-CLEAs were highly stable after the recycling process, retaining about 60% of the original activity after the tenth cycle. *Ha*SGNH1 was also immobilized on magnetite Fe_3_O_4_ nanoparticles for easy separation and fast recovery [20, 37]. To obtain magnetic *Ha*SGNH1-CLEAs (mCLEA-*Ha*SGNH1), *Ha*SGNH1 was co-precipitated using Fe_3_O_4_ nanoparticles and cross-linked using glutaraldehyde. SEM images of mCLEA-*Ha*SGNH1 confirmed the formation of small tiny globular structures with a size of 0.2 to 0.3 μm (Additional file 1: Fig. S4C). mCLEA-*Ha*SGNH1 retained about 65% of its original activity after the seventh cycle. Similar results have also been observed using other proteins [20, 30, 37]. In summary, immobilization of *Ha*SGNH1 was effectively carried out using three different approaches (*Ha*SGNH1-CLEA, *Ha*SGNH1-Arg-CLEA, and mCLEA-*Ha*SGNH1), and there could be exploited to facilitate *Ha*SGNH1 use in industrial applications.

### Synthesis of butyl and oleic esters

**Fig. 8:**
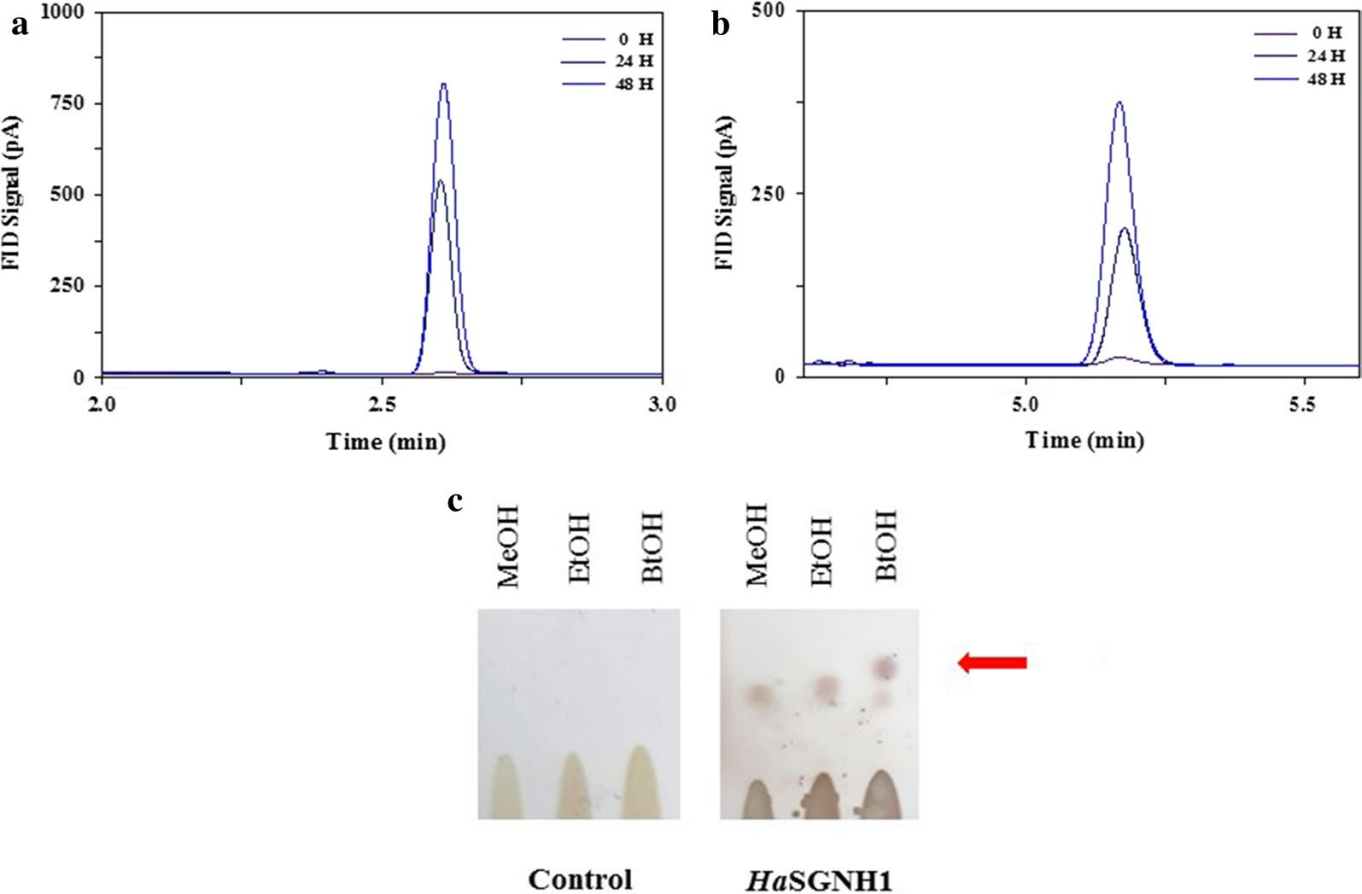
Butyl and oleic esters synthesis using *Ha*SGNH1-CLEAs. **a** Gas chromatographic analysis of **a** butyl acetate and **b** butyl butyrate formation. In **a**, **b**, each curve represents the formation of butyl acetate and butyl butyrate formation at 0 h, 24 h, and 48 h, respectively. **c** Thin layer chromatography (TLC) analysis of the formation of oleic acid esters. Oleic acid was incubated with methanol (MeOH), ethanol (EtOH), and butanol (BtOH) and CLEAs-*Ha*SGNH1

Utility of *Ha*SGNH1-CLEA as a biocatalyst for butyl and oleic esters was explored based on the high recyclability. Butyl esters are valuable fuel sources that can be processed with other petroleum-based products. In addition, butyl esters show interesting properties like a high boiling point, low viscosity, and low temperature behaviors for food and cosmetic industries [38, 39]. *Ha*SGNH1 could be suitable for butyl esters production because it prefers short-chain length substrates, and butyl esters have been successfully synthesized by several other lipases [40, 41]. As shown in Fig. 8a, *Ha*SGNH1 could effectively synthesize butyl acetate from 1-butanol and acetic acid based on a gas chromatogram, which is comparable to other enzymes [42, 43]. In addition, *Ha*SGNH1 showed significant catalytic efficiency for butyl butyrate synthesis (Fig. 8b). The esters of long-chain fatty acids are widely used as a biodiesel and many attempts have been made to identify novel lipases suitable for the production of these esters [44, 45]. Furthermore, *Ha*SGNH1 could catalyze the formation of oleic acid esters using the substrates—oleic acid and alcohols (methanol, ethanol, and butanol). Methyl, ethyl, and butyl oleate biosynthesis were observed using thin-layer chromatography (TLC) (Fig. 8c). Gas chromatography–mass spectrometry (GC/MS) analysis also confirmed the formation of butyl acetate (2.736 min), butyl butyrate (4.938 min), and oleic acid butyl ester (20.508 min) (Additional file 1: Fig. S5). The findings suggest that *Ha*SGNH1 could be used to prepare fatty acid methyl ester (FAME) biodiesels. Collectively, *Ha*SGNH1 displayed a promising ester synthesis performance, and it could be used for various applications in the cosmetics, pharmaceutical, and food industries.

## Conclusion

Although SGNH-type lipases have attracted great interest due to their potential applications in a wide range of industrial fields including biodiesel production and ester synthesis, there is still little information about this family in psychrophilic bacterium. Here, we analyzed the genome of the recently sequenced psychrophilic/halophilic bacterium *H. arcticus*. We reported the characterization and immobilization a novel promiscuous cold-adapted SGNH-type lipase (*Ha*SGNH1) and its application in esters and biodiesel synthesis. The remarkable properties of *Ha*SGNH1 could make it a promising candidate for a wide range of applications in the chemical and biofuel industries [46, 47]. To our knowledge, this is the first study to clearly indicate that cold-adapted SGNH-type lipase could be used to catalyze butyl and oleic esters synthesis. This finding could be used in the food, cosmetics, pharmaceutical, and biofuel industries.

## Materials and methods

### Reagents

Restriction endonucleases and other DNA modifying enzymes were obtained from New England BioLabs (Ipswich, MA, USA). DNA purification kits and protein purification columns were obtained from Qiagen Korea (Daejon, Korea) and GE Healthcare Korea (Seoul, Korea). All other highly analytical grade reagents were purchased obtained from Sigma-Aldrich Korea (Yongin, Korea).

### Bioinformatic analysis

The primary sequences of *Ha*SGNH1 and other bacterial esterase/lipases were retrieved from the NCBI database. A phylogenetic tree was built using the neighbor-joining (NJ) method in the MEGA 7.0 software package [48]. Multiple sequence alignments were carried out using Clustal Omega [49] and ESPript [50]. A structural model of *Ha*SGNH1 was generated using the TesA crystal structure from *P. aeruginosa* (PDB code: 4JGG, 39% sequence identity) as a template on the SWISS-MODEL server. All graphical representations were prepared using the PyMOL software.

### Preparation of *Ha*SGNH1

*H. arcticus* (KCTC 42129, Korean Collection for Type Cultures) was cultured in Marine Agar 2216 and genomic DNA was purified using a DNeasy Tissue and Blood Kit (Qiagen, USA). The open reading frame of the *Ha*SGNH1 gene was amplified from *H. arcticus* genomic DNA using a pair of primers, (forward primer 5′-TAA ATC GCT AGC ATG AGT GCT CGC GTT-3′ and reverse primer 5′-CAT GCA CTC GAG CTA TTC TTG TGT CTG-3′). The PCR product was cloned into pET-21a and transformed into *E. coli* BL21 (DE3) to express *Ha*SGNH1 with an N-terminal hexahistidine tag. Site-directed mutagenesis of *Ha*SGNH1 was conducted using the Quik-change site-directed mutagenesis method (Stratagene, CA, USA). All variants (S18A, N83L, M86E, M86R, R87L, F131A, and I173F) were purified using the same method as wild-type *Ha*SGNH1.

Transformed *E. coli* BL21 (λDE3) cells were grown in LB medium until the OD_600_ reached 0.6 to 0.7. After induction with 1 mM isopropyl-β-d-1-thiogalactoside **(**IPTG) for 18 h at 20 °C, bacterial cells were centrifuged at 6000 rpm for 20 min at 4 °C and resuspended in lysis buffer (20 mM Tris–HCl, 100 mM NaCl, 20 mM imidazole, and 1 mM EDTA, pH 7.4). The cell suspension was lysed using sonication, and cell debris was centrifuged at 20,000 rpm for 30 min. The supernatants were loaded onto a HisTrap HP column in an AKTA Prime Plus (GE healthcare, USA). The recombinant *Ha*SGNH1 protein was eluted using an imidazole gradient from 50 to 200 mM. The resulting fractions were buffer-exchanged into a storage buffer (20 mM Tris–HCl, and 1 mM EDTA, pH 8.0). Protein purity and the molecular weight were confirmed using Coomassie brilliant blue (CBB)-stained sodium dodecyl sulfate–polyacrylamide gel electrophoresis (SDS-PAGE). Protein concentrations were determined using a Bio-Rad Protein assay kit (IL, USA) with bovine serum albumin (BSA) as a standard. The final yield of active *Ha*SGNH1 lipase was ~ 5 mg/g cell dry weight (CDW). The OD_600_ values were converted to cell dry weight using an OD_600_/dry cell weight relationship for *E. coli* (1.0 OD_600_ = 0.32 gDW/L) [51].

### Biochemical characterization of *Ha*SGNH1

Activity staining was performed by using native-PAGE incubated with Coomassie Brilliant Blue R-250 and 4-methylumbelliferone (4-MU) acetate [52, 53]. Hydrolysis of 4-MU acetate or phosphate in an Eppendorf tube containing *Ha*SGNH1 was also observed with strong fluorescence in an UV illumination box. Purified *Ha*SGNH1 was applied to HiPrep Sephacryl S-200R column at a flow rate of 0.5 mL min^−1^ for gel filtration analysis. Far-UV CD spectra were recorded from 190 to 260 nm using a Jasco J-815 spectropolarimeter (Jasco, Japan). Data collection was carried out in a 1-mm path-length cell with a 0.5 nm bandwidth, 1 s response time, and 100 nm min^−1^ scan speed. Thermal unfolding was monitored using CD signals with a thermostatic cell holder from 15 °C to 80 °C at 222 nm.

Substrate specificities of *Ha*SGNH1 and its variants were determined using *p*-nitrophenyl (*p*-NP) esters and naphthyl ester derivatives [54]. The standard assay solution included 50 μM substrates in 20 mM Tris–HCl (pH 8.0) with 0.5 μg *Ha*SGNH1, and the assay was run for 30 s at 25 °C. The influence of pH on the activity of *Ha*SGNH1 was investigated using buffers in a wide pH range from 3 and 10. The effects of chemicals (ethanol, isopropanol, Tween 20, Triton X-100, urea, SDS, NaCl, and glycerol) on *Ha*SGNH1 activity were determined using relative activities after a 1-h incubation. All assays described above were carried out with *p*NP-C_4_ as a substrate and an Epoch 2 Microplate spectrophotometer (BioTek, VT, USA). The enzyme activity of *Ha*SGNH1 in buffer alone was defined as 100%.

The optimum thermostability was investigated at different temperature ranging from 0 to 25 °C. The effect of temperature on *Ha*SGNH1 activity was assayed over a temperature ranging from 15 to 100 °C. The percent residual activity with respect to the initial activity was evaluated for thermal stability. Freeze-thawing experiments were carried out for 20 cycles between freezing (− 70 °C for 1 h) and thaw (25 °C for 1 h) steps.

For colorimetric assays, *Ha*SGNH1 was included in a phenol red-containing substrate solution (tertiary butyl acetate, α-terpinyl acetate, linalyl acetate, glyceryl tributyrate, glyceryl trioleate, fish oil, and olive oil). Quantification of acetic acids released from the hydrolysis of various substrates was quantitatively determined using an acetic acid kit (K-ACET, Megazyme, USA) according to the manufacturer’s instructions. The kinetic parameters of *Ha*SGNH1 were determined using various concentrations of *p*-NA, *p*-NB, and *p*-NH, and each initial velocity was calculated by averaging three independent measurements. All these data were fitted to the Michaelis–Menten equation, and V_max_, *K*_m_, *k*_cat_, and *k*_cat_*/Km* were calculated using double reciprocal plots.

### Immobilization of *Ha*SGNH1

For the preparation of cross-linked enzyme aggregates (CLEAs), 0.5 mg mL^−1^ of *Ha*SGNH1 was co-precipitated by 80% (w/v) ammonium sulfate at 4 °C. Crosslinking was performed using dropwise addition of glutaraldehyde to a 25 mM final concentration at room temperature. After an overnight incubation and centrifugation at 15,000*g* for 10 min, the pellet (*Ha*SGNH1-CLEA) was resuspended and washed repeatedly until no activity was observed in the supernatant. Addition of arginine and magnetic Fe_3_O_4_ nanoparticles was carried out as previously described [20, 37]. Scanning electron microscope images were obtained at various magnifications (50,000 to 100,000×) using a Carl Zeiss SUPRA 55VP. For the recycling process, three forms of immobilized *Ha*SGNH1 (*Ha*SGNH1-CLEA, *Ha*SGNH1-Arg-CLEA, and mCLEA-*Ha*SGNH1) were reused for the next cycle after an extensive washing process.

### Synthesis of butyl and oleic esters

For butyl acetate synthesis, dried CLEA-*Ha*SGNH1 was incubated with 1 M 1-butanol and 1 M acetic acid in hexane with a total volume of 1 mL [20, 55]. 2 µL of the sample was directly analyzed with a HP-5 capillary column using gas chromatography (Agilent 7890, Agilent Technologies, Santa Clara, CA, USA). The initial oven temperature was set to 35 °C (1 min) and increased to 160 °C at a 10 °C/min ramping rate. The temperatures of the injector and detector were set at 190 °C. For oleic acid esters synthesis, dried CLEAs-*Ha*SGNH1 was incubated with 1 M methanol, ethanol, 1-butanol and 2 M oleic acid in hexane with a total volume of 1 mL [20]. The samples were directly analyzed using thin-layer chromatography (TLC) with hexane:ether:formic acid (80:15:1, v/v) as a mobile phase. TLC plates were dipped into ethanol:sulfuric acid (9:1, v/v) solution and heated until the bands were fully developed [56–58]. For the identification of resulting compounds, gas chromatography/mass spectrometry (GC/MS) was performed by 6890 Agilent GC with 5973 mass selective detector. The compounds were then verified using Wiley mass spectra library [59].

### Statistical analysis

All experiments and assays were performed in triplicates and error bars represent the standard deviation. Data were analyzed using the two-tailed unpaired Student’s *t* test and analysis of variance (ANOVA) in GraphPad Prism software. The statistical significance was set at the level of *p* < 0.05.

## Supplementary information

**Additional file 1.** Additional figures.

## Data Availability

All data generated or analyzed during this study are included in this published article and its additional files.

## Abbreviations

ANOVA: Analysis of variance
BSA: Bovine serum albumin
CD: Circular dichroism
CE: Carbohydrate esterase
FAME: Fatty acid methyl ester
GC/MS: Gas chromatography–mass spectrometry
GDSL: Gly-Asp-Ser-Leu
GTB: Glyceryl tributyrate
*k*_cat_: Turnover number
*K*_*M*_: Michaelis–Menten constant
*k*_cat_*/K*_*m*_: Catalytic efficiency
4-MU: 4-Methylumbelliferone
1-NA: 1-Naphthyl acetate
2-NA: 2-Naphthyl acetate
NJ: Neighbor-joining
*p*-NA: *p*-Nitrophenyl acetate
*p*-NB: *p*-Nitrophenyl butyrate
*p*-NH: *p*-Nitrophenyl hexanoate
SDS-PAGE: Sodium dodecyl sulfate–polyacrylamide gel electrophoresis
TAE: Tertiary alcohol ester
TLC: Thin-layer chromatography
UV: Ultraviolet
V_max_: Maximum velocity
