# Supplementary material for: Biodiesel and flavor compound production using a novel promiscuous cold-adapted SGNH-type lipase (HaSGNH1) from the psychrophilic bacterium Halocynthiibacter arcticus
Source: Biotechnol Biofuels. 2020 Mar 16;13:55. doi: 10.1186/s13068-020-01696-x (PMC7074997; doi:10.1186/s13068-020-01696-x)

**Additional Figures Legends**

**Figure S1. Characterization of *Ha*SGNH1.**

(A) Size-exclusion chromatographic analysis of *Ha*SGNH1. The column was calibrated with *β*-amylase (200 kDa), IgG (150 kDa), BSA (66 kDa), carbonic anhydrase (29 kDa), and cytochrome C (12 kDa). (B) Far-UV CD analysis of *Ha*SGNH1 and *Nm*SGNH1. (C) Thermal unfolding of *Ha*SGNH1 was monitored by CD at 222 nm from 10 to 80 °C. (D) Chemical stability of *Ha*SGNH1. (E) Effects of NaCl and glycerol on *Ha*SGNH1 activity. (F) Effects of urea on the activity of *Ha*SGNH1. In these experiments, *Ha*SGNH1 was incubated for 1 hour prior to the hydrolysis of *p-*NB.

**
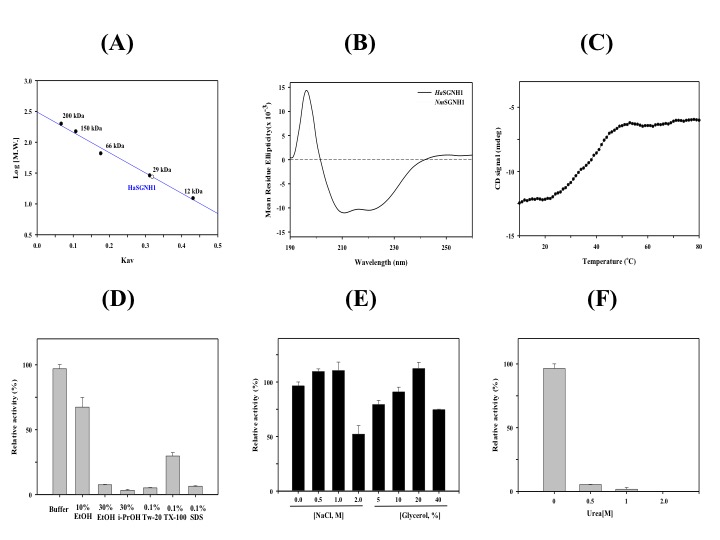
**

**Figure S2. Enzymatic properties and structural analysis tion of *Ha*SGNH1.**

The hydrolysis of (A) tertiary alcohol esters (*t*-BA: *tert*-butyl acetate; LA: linalyl acetate; *α*TA: α-terpinyl acetate) and (B) lipids (GTB: glyceryl tributyrate; GTO: glyceryl trioleate; FO: fish oil; OO: olive oil). Substrate binding regions of (C) *Ha*SGNH1, (D) EstA from *Pseudoalteromonas* sp. 643A (PDB: 3HP4), and (E) TesA from *P. aeruginosa* (PDB: 4JGG) were shown.


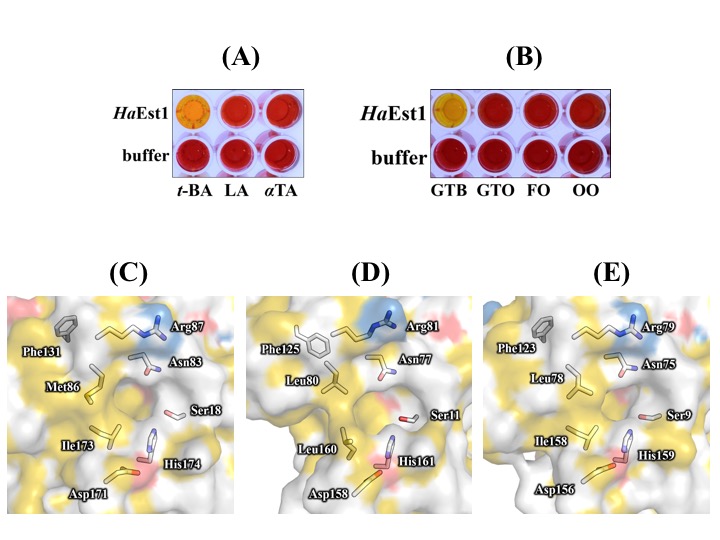


**Figure S3. Kinetic analysis of *Ha*SGNH1 and its variants.** *Ha*SGNH1 hydrolysis of (A) *p*NA, (B) *p*NB, and (C) *p*NH was investigated at a wide range of concentrations. Michaelis-Menten plot of the hydrolysis of *p*NB by (A) M86R, (B) M86E, and (C) R87L.


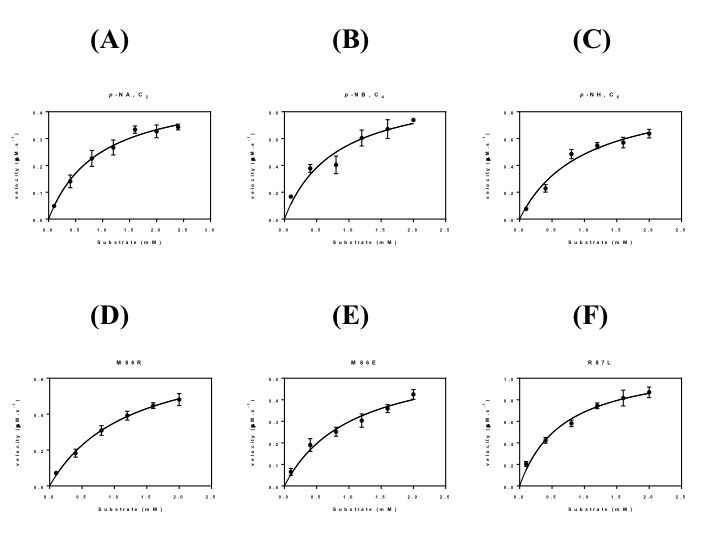


**Figure S4. Immobilization of *Ha*SGNH1.** Field emission scanning electron microscopic (FE-SEM) images and durability of immobilized *Ha*SGNH1 were investigated for (A) cross-linked enzyme aggregates (CLEAs), (B) cross-linked enzyme aggregates with L-arginine (L-Arg-CLEAs), and (C) magnetic cross-linked enzyme aggregates (mCLEAs). For FE-SEM images, 25,000X (left) and 100,000X (middle) magnifications. Scale bars are (A) 1 μm, and (B) 200 nm. Reusability of all three immobilized forms was also investigated (right).


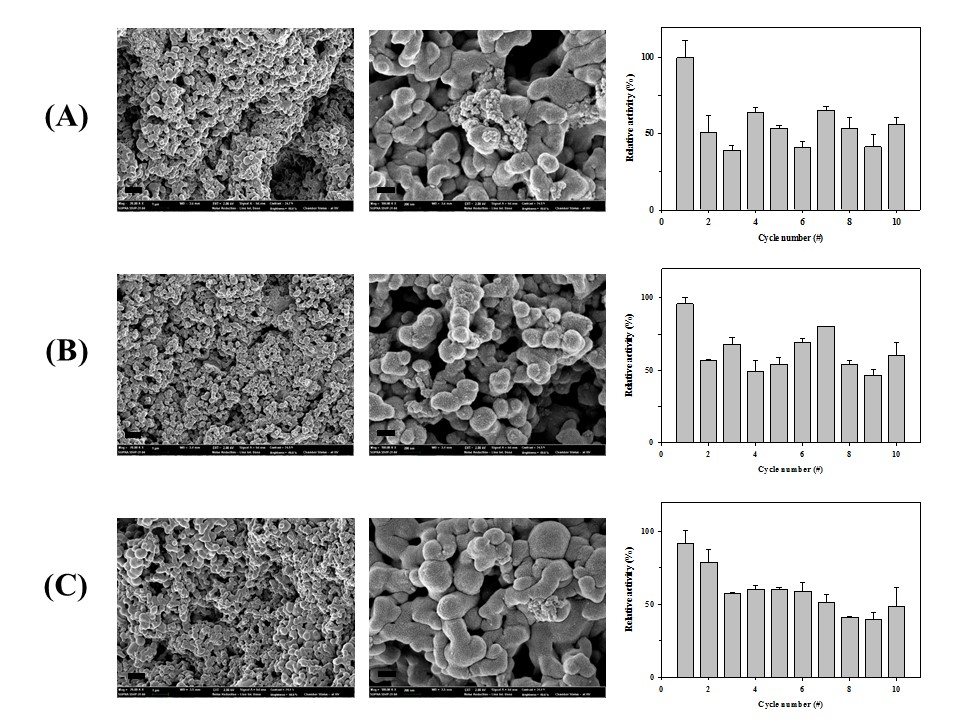


**Figure S5. GC/MS analysis.** Gas chromatography/mass spectrometric (GC/MS) analysis of esters prepared by CLEAs-*Nm*SGNH1. The identified peaks were assigned to butyl acetate (A), butyl butyrate (B), and oleic acid butyl ester (C).


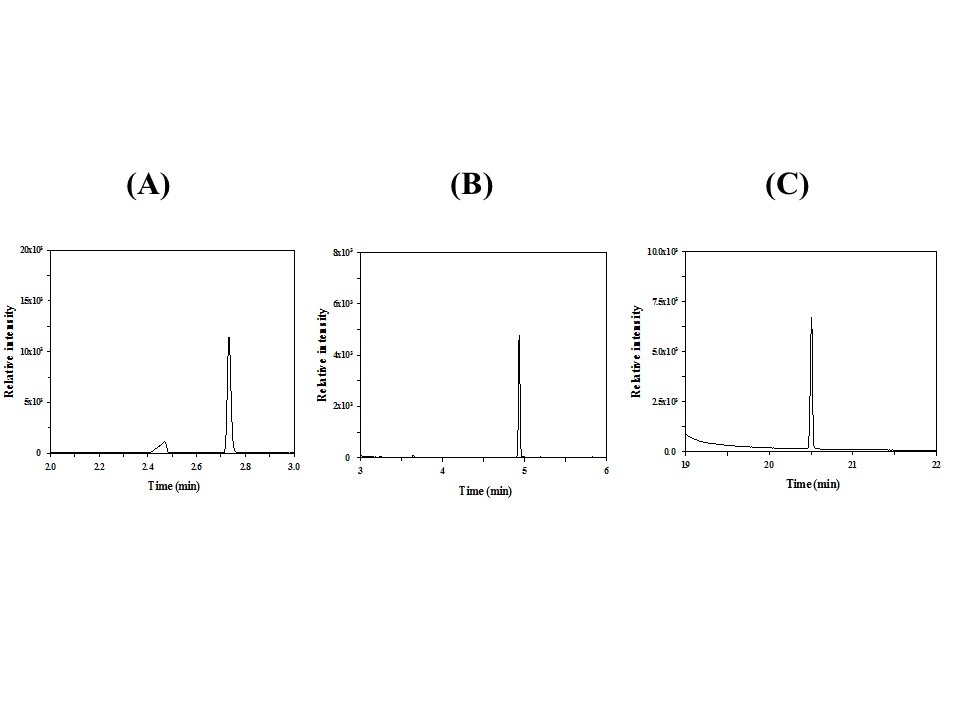

Supplement: Supplementary file 1 — Additional file 1. Additional figures. [file 13068_2020_1696_MOESM1_ESM.docx]
